# Ancient diversity of Afrotropical *Microborus*: three endemic species – not one widespread

**DOI:** 10.3897/zookeys.710.14902

**Published:** 2017-10-19

**Authors:** Bjarte H. Jordal

**Affiliations:** 1 Natural History Museum, The University Museum, University of Bergen, NO-5007 Bergen, Norway

**Keywords:** Curculionidae, Scolytinae, Hexacolini, *Microborus*, molecular phylogeny, Africa, Madagascar

## Abstract

The primarily Neotropical genus *Microborus* Blandford is represented with three species in Africa and Madagascar. The previously recorded species from this region, *M. boops* Blandford, is a Neotropical species restricted to Central America and is likely not found in the Afrotropics. The previously recognised species in western parts of Africa is *M. camerunus* (Eggers) and is resurrected from synonymy under *M. boops.* Molecular and morphological data revealed a second species of this complex in Madagascar, *M. brevisetosus* Jordal. Another new species, *M. angustus* Jordal, co-occurs with *M. camerunus* in Cameroon. Substantial genetic divergence indicate that *Microborus* was established in the Afrotropical region long before human transport across oceans. A key to Afrotropical species is provided.

## Introduction

**Figures 1–2.**
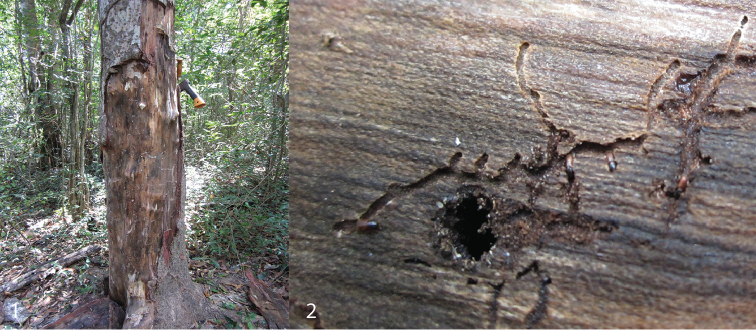
Typical host plant condition for species of *Microborus*. **1** Standing *Stereospermum* tree with thick bark, with attacks of ambrosia beetles, cossonine weevils and *Microborus* in the lower bole (Ankarafantsika NP, Madagascar). **2** Inner side of bark with tunnels made by *M. brevisetosus* Jordal, starting from the entrance hole of *Euplatypus madagascariensis* (Schedl).

*Microborus* Blandford, 1897 is a largely Neotropical genus consisting of eight known species, with one of these also recorded from the Afrotropical region. Species are generally small in size, but are often taken from very thick bark of large tree trunks (Wood 2007). Their breeding biology is unusual in that nests are initiated via the entrance opening of a much larger bark or ambrosia beetle species and mines away from their host gallery just inside the entrance (Figs 1–2).

Previous classifications have placed *Microborus* in the scolytine tribe Hexacolini (previously Ctenophorini, see Alonso-Zarazaga and Lyal 2009). Recent molecular phylogenies have nevertheless questioned the relationship to *Scolytodes* Ferrari and other hexacoline genera, and instead pointing towards a relatively isolated position in Scolytinae. This is a very old genus that apparently diverged from all other extant lineages more than 100 Ma (Jordal and Cognato 2012) and which experienced very little morphological change since its time of origin (Cognato and Grimaldi 2009).

The supposedly broad distribution of *Microborus boops* Blandford in the Neotropical and Afrotropical regions has been inferred as a recent introduction to Africa and Madagascar (Wood 1982). Using integrated morphological and molecular data, the Afrotropical fauna is revised, including discovery of two new species, and rejection of a globally widespread distribution in *M. boops*.

## Materials and methods

Specimens included were collected during the author’s field expeditions to Cameroon (2007) and Madagascar (2015). Deposition of typematerial are indicated by the following acronyms: BMNH, Natural History Museum London; CAS, California Academy of Science; NHMW, Naturhistorisches Museum Wien; ZMBN, University Museum of Bergen (formerly Zoological Museum, Bergen).

DNA was extracted from whole specimens, of which the macerated body remains were mounted on slides or glued on a pinned card. Six gene fragments were amplified: COI, EF-1α, 28S, CAD, ArgK and PABP1 (Jordal et al. 2011; Pistone et al. 2016). Sequences were concatenated for combined phylogenetic analyses using maximum likelihood and maximum parsimony in PAUP* (Swofford 2002).

Morphological examination of internal or hidden characters such as flight wings, proventriculus and male genitalia was only made for one species that had sufficient specimens available.

## Taxonomy

### Microborus

Taxon classificationAnimaliaColeopteraCurculionidae

Blandford, 1897

#### Type species.

*Microborus boops* Blandford, 1897

#### Diagnosis.

Small slender species with pronotum laterally costate, anteriorly unarmed; procoxae separated by broad prosternal process; eyes large, approximate below; antennal club globular without sutures, funicle 6-segmented; elytral interstriae 7 sharply raised on declivity and curved towards elytral interstriae 9 to form a postero-lateral costa on declivity.

### Microborus angustus

Taxon classificationAnimaliaColeopteraCurculionidae

Jordal sp. n.

http://zoobank.org/B02BF8A3-3D75-4518-BDA0-617A3D748DFC

Figs 3–4 , 9–11 , 18–20

#### Type material examined.

Holotype: Cameroon, Mt. Cameroon south slope, 1600m alt., *Ficus* branch, B. Jordal 28xi-8 [28. Nov. 2007]. ZMBN/ENT_Scol4932. Paratypes (8): same data as HT (ZMBN/ENT_Scol4933-4940). (GIS: 4.12, 9.16). All types deposited in ZMBN.

#### Diagnosis.

A very elongated, almost black species, with impressed elytral striae and a distinct costate rim along the postero-lateral margin of elytral declivity.

#### Description

**(male and female).** *Length* 1.3–1.5 mm, 2.7–2.8 × longer than wide.*Colour* dark brown, almost, black, legs and antennae light brown.

*Head.* Eyes separated above by 1.4 × their width. Frons reticulate and deeply punctured, smooth and shiny at level of antennal insertion, vestiture consisting of a few scant fine setae.

*Pronotum* smooth, shiny, with densely placed punctures.

*Elytra* with all striae impressed, punctures deep, subconfluent; interstriae as wide as striae, with very fine irregularly spaced punctures; postero-lateral rim sharply elevated with 3–6 sharp granules. Vestiture consisting of few long, fine, erect golden setae.

*Legs.* Protibiae with three lateral teeth (embedded denticles), and one additional tooth just above the inner mucro; posterior face smooth.

*Ventral vestiture* simple, on ventrites very fine, short setae.

*Wings* typical for weevils, costa with two setae close to each other near base, and one seta two-thirds the distance towards the stigmal patch; anal field missing, posterior margin with long fine setae; stigmal patch with two short, sharp setae, each on a small tubercle.

*Proventriculus* with apical plate well developed, median suture wide open, sutural teeth long and sharp, apical teeth and marginal bristles missing, closing teeth long and prominent, >10 large femoral teeth.

*Male genitalia* very simple, spiculum gastrale not present, no distinction between apophyses and aedeagal body, internal sac with granulated surface, tegmen open dorsally, gradually broader ventrally with a short manubrium.

**Figures 3–8.**
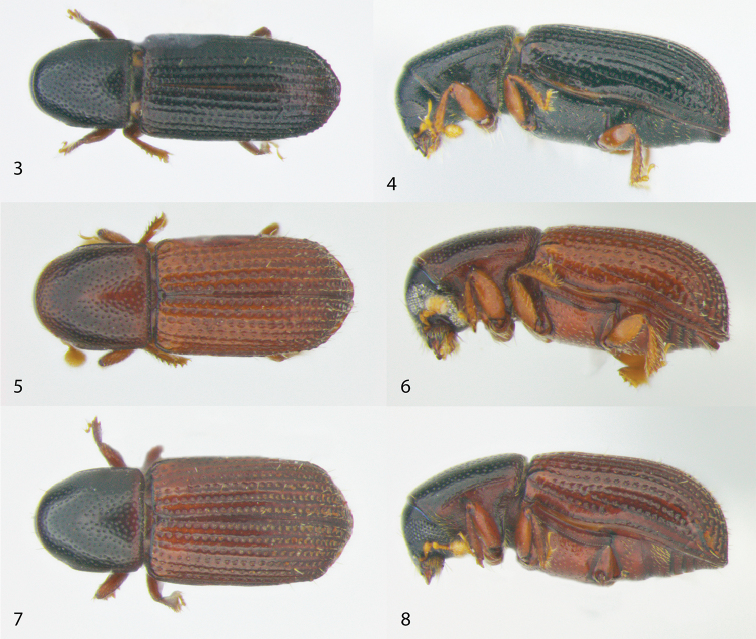
Dorsal and lateral view of the Afrotropical species of *Microborus*. **3–4** *M. angustatus* **5–6** *M. brevisetosus* **7–8** *M. camerunus*.

**Figures 9–17.**
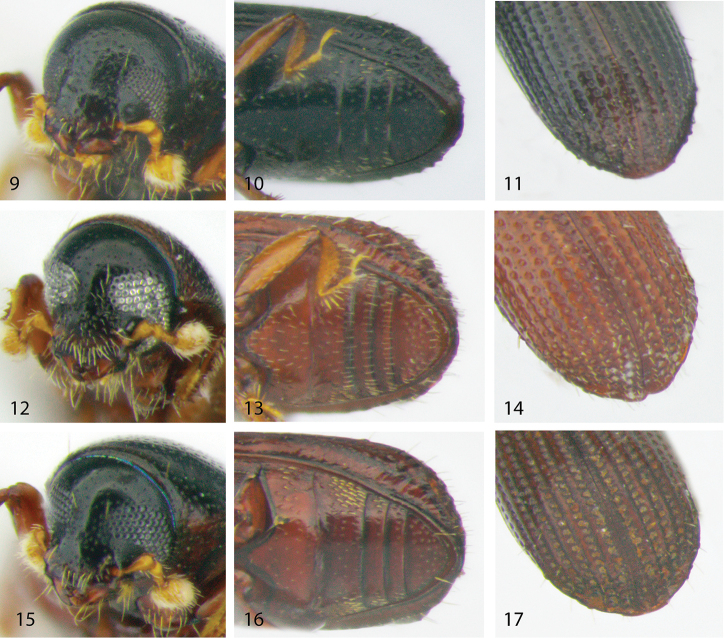
Head, venter and declivity of the Afrotropical species of *Microborus*. **9–11** *M. angustatus* **12–14** *M. brevisetosus* **15–17** *M. camerunus*.

**Figures 18–20.**
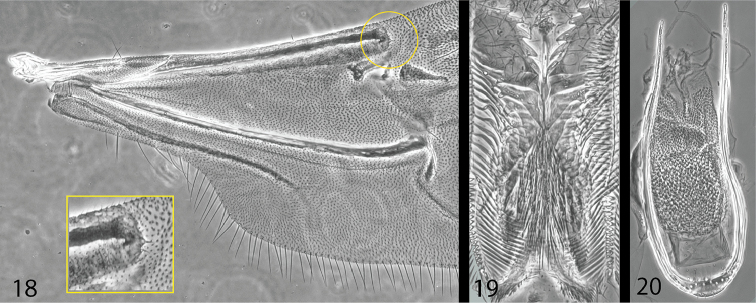
Internal features of *Microborus angustus*. **18** wing base **19** proventriculus **20** aedeagus.

#### Etymology.

Latin adjective *angustus*, meaning narrow. This is the most elongated species in the genus in the Afrotropical region.

#### Distribution and biology.

Only known from the type locality. It was taken together with *M. camerunus* (Eggers, 1919) under thick bark of a fallen *Ficus* tree. Both species used entrance holes made by *Xyleborus principalis* Eichhoff, 1878.

### Microborus brevisetosus

Taxon classificationAnimaliaColeopteraCurculionidae

Jordal sp. n.

http://zoobank.org/17AC91AF-2FA6-46B3-8D16-CB8FC53E9FA4

Figs 5–6 , 12–14

#### Type material examined.

Holotype: Madagascar, Boeny province, Ankarafantsika NP, 200 m alt. GIS [-16.264, 46.828], ex *Stereospermum* standing tree, 8.May.2015, B. Jordal leg. (ZMBN/ENT_Scol4929). Paratypes (2): same data as HT (ZMBN/ENT_Scol4930). Madagascar, Forêt de Tsimembo, 11.0 km 346° NNW Soatana, GIS [-18.995, 44.444], 21.Nov.2001, B. Fischer, BLF4508, (1). HT and one PT in ZMBN, 1 PT in CAS.

#### Diagnosis.

Distinguished from *M. camerunus* (Eggers) by the much more abundant short setae in the lower frons, nearly moustache-like on epistoma, ventrites 2–4 with regular transverse rows of fine recumbent setae, and posterior face of protibiae with 3–5 sharp granules. It is distinguished from *M. boops* by the short and bristle-like setae on elytral interstriae on declivity.

#### Description

**(male and female).** *Length* 1.4 mm, 2.6 × longer than wide. *Colour* reddish brown, pronotum darker.

*Head.* Eyes separated above by 0.6 × their width. Frons reticulated and lightly punctured, protruding slightly below eyes, vestiture consisting of >50 short setae, longer between eyes and some on epistoma.

*Pronotum* smooth, shiny, with densely placed puntures.

*Elytra* with striae impressed, punctures deep, spaced by distance equal to their diameter; interstriae about half as broad as striae, with very fine irregularly spaced punctures; postero-lateral interstrial rim slightly elevated with 2–3 blunt granules. Vestiture consisting of a few longer, erect, golden setae on discal interstriae, with densely placed, short, stiff setae on declivity.

*Legs.* Protibiae with three lateral teeth (embedded denticles), and one additional tooth just above the inner mucro; posterior face rough.

*Ventral vestiture* simple, on ventrites 1–4 consisting of fine, long recumbent setae forming transverse rows.

#### Etymology.

Latin adjectives *brevis*, meaning short, and *setosus*, meaning bristly, referring to the very short stiff interstrial setae on the elytral declivity.

#### Distribution and biology.

Madagascar: Boeny, Melaky, Diana and Analanjirofo provinces. Specimens were examined only from the western part of the island. It is presumed that Schedl’s reported specimens from the east and north of the island are conspecific. The collection from Ankarafantsika (Fig. 1) was taken from brood galleries under thick bark of a standing *Stereospermum* tree, iniated via the entrance holes of *Euplatypus madagascariensis* (Chapuis, 1865).

### Microborus camerunus

Taxon classificationAnimaliaColeopteraCurculionidae

(Eggers, 1919) stat. n.

Figs 7–8 , 15–17

Pseudocrypturgus camerunus Eggers, 1919: 236, original description.
Microborus camerunus (Eggers, 1919): synonymized with M. boops Blandford, 1897, by Wood (1982), here resurrected.

#### Type material examined.

Holotype of *Pseudocrypturgus camerunus* Eggers (NHMW). Holotype of *Microborus boops* Blandford (BMNH).

#### Diagnosis.

Distinguished from *M. brevisetosus* and *M. boops* by the smooth and glabrous frons, the glabrous central area of the ventrites, the smooth posterior face of the protibiae, subconfluent strial punctures, and the slightly stouter body shape.

#### Description

**(male and female).** *Length* 1.5 mm, 2.4 × longer than wide. *Colour* reddish brown, pronotum darker.

*Head.* Eyes separated above by 0.7 × their width. Frons smooth, shiny and lightly punctured, vestiture consisting of <10 short setae on epistoma and 2 longer setae between eyes.

*Pronotum* smooth, shiny, with densely placed puntures.

*Elytra* with striae impressed, punctures deep, subconfluent; interstriae about as broad as striae, with very fine irregularly spaced punctures; postero-lateral (interstrial) rim slightly elevated with 2–3 blunt granules. Vestiture consisting of scattered erect, golden setae on discal interstriae, somewhat shorter on declivity.

*Legs.* Protibiae with three lateral teeth (embedded denticles), and one additional tooth just above the inner mucro; posterior face smooth.

*Ventral vestiture* simple, on ventrites consisting of a few irregularly placed short setae close to the lateral margins.

#### Distribution and biology.

Known from Ghana, Cameroon and Congo. New record: Cameroon, Mt. Cameroon south slope, 1600 m, GIS: [4.12, 9.16], *Ficus* branch, B. Jordal 28xi-8 [28. Nov. 2007] (ZMBN/ENT_Scol4931, 4941). It was taken together with *M. angustus* under thick bark of large fallen *Ficus* tree (see above).

##### Key to the Afrotropical species of *Microborus*

**Table:** 

1	Nearly black, 2.8 × longer than wide; elytral interstria 7 on declivity sharply raised and almost serrated, with 4–5 sharp tubercles. Cameroon	***M. angustus* Jordal**
–	Reddish brown, 2.4–2.6 × longer than wide, elytral interstria 7 on declivity raised, tubercles obscure	**2**
2	Strial punctures on elytra separated on average by 0.7–1 × their diameter; ventrites 1–4 with recumbent short setae in transverse row along the posterior edge; frons with >50 small setae, rather dense and moustache-like on epistoma; posterior face of protibiae with 2–5 sharp granules. Madagascar	***M. brevisetosus* Jordal**
–	Strial punctures on elytra confluent or nearly so; ventrites with few short setae scattered along their lateral sides; frons with <10 scattered setae; posterior face of protibiae smooth. Ghana-Congo	***M. camerunus* (Eggers)**

##### Molecular data on Afrotropical species

**Figure 21.**
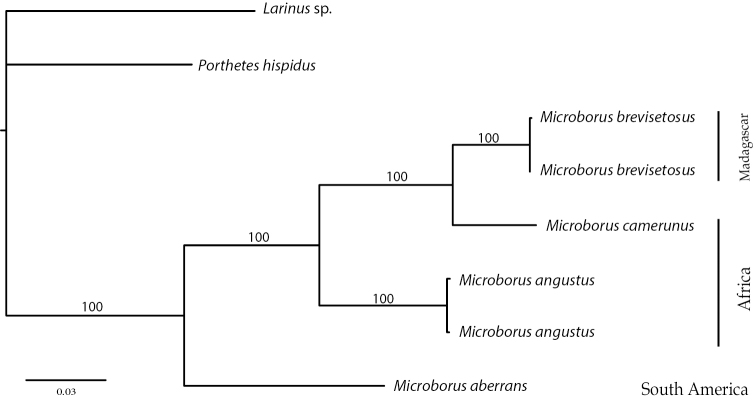
Maximum likelihood phylogeny based on six gene fragments and a GTR model of gene evolution. Tree topology and bootstrap values were identical in the maximum parsimony analysis (L = 1657, CI = 0.85, RI = 072).

Gene sequences obtained via PCR are listed by their genbank accession numbers in Table 1. Maximum likelihood and maximum parsimony provided consistent results across analyses, with all nodes maximally supported (Fig. 21). The three Afrotropical species formed a group separate from the single Neotropical species included, *M. aberrans*. *Microborus angustus* was furthermore clearly distinct from the sympatric *M. camerunus* that grouped closely with *M. brevisetosus*, suggesting a role for allopatric divergence prior to the co-existence of *M. angustatus* and *M. camerunus*. Given the limited global scope in this study, it cannot be ruled out that the Afrotropical (or the Neotropical) fauna experienced two origins for this genus.

**Table 1.** Samples included for DNA analyses.

Species	Voucher	Locality	COI	EF-1α	28S	CAD	ArgK	PABP1
*Larinus sp.*	ClLar01	Russia: Vladivostok	HQ883622	HQ883707	HQ883541	HQ883773	HQ883854	KX160752
*Porthetes hispidus*	MoPor01	South Africa: Kokstad	HQ883666	HQ883737	HQ883577	HQ883805	HQ883895	KX160765
*Microborus aberrans*	CtMic07	Brasil: Manaus	MF803724	MF803728	MF803715	MF803720	MF803717	MF803732
*Microborus angustus*	CtMic03	Cameroon: Mt. Cameroon	HQ883645	–	HQ883560	HQ883788	HQ883874	KU041929
*Microborus angustus*	CtMic04	Cameroon: Mt. Cameroon	MF803721	MF803725	MF803713	MF803718	MF803716	MF803729
*Microborus brevisetosus*	CtMic01	Madagascar: Forêt de Tsimembo	HQ883645	HQ883724	HQ883559	HQ883787	–	–
*Microborus brevisetosus*	CtMic06	Madagascar: Ankarafantsika NP	MF803723	MF803727	–	MF803719	–	MF803731
*Microborus camerunus*	CtMic05	Cameroon: Mt. Cameroon	MF803722	MF803726	MF803714	–	–	MF803730

Despite high morphological similarity, *M. brevisetosus* and *M. camerunus* differed by 15.3–16.1 % at COI. An average divergence of 2.4–3.3% at five nuclear loci leave no doubt about each species validity. The largest nuclear variation was found in 28S (3.9%), a substantial difference for morphologically similar taxa (see e.g. Jordal and Kambestad 2014). Guided by the molecular data, a search for consistent morphological differences was found in the frons, elytral declivity and the venter of these beetles. Hence, the overall similarity that has led previous researchers to synonymise *M. camerunus* with *M. boops* (Wood 1982), emphasizes the need for careful consideration of possible semi-cryptic character differences. The low rate of change in morphological characters for the genus as a whole, as documented by the close similarity to the mid-Cretaceous fossil *M. inertus* Cognato & Grimaldi, 2009 (see Cognato and Grimaldi 2009), makes it advisable to base new synonymies on genetic data and rigorous morphological examination.

## Supplementary Material

XML Treatment for Microborus

XML Treatment for Microborus angustus

XML Treatment for Microborus brevisetosus

XML Treatment for Microborus camerunus
